# Evidence of the causal role of human papillomavirus type 58 in an oropharyngeal carcinoma

**DOI:** 10.1186/1743-422X-10-334

**Published:** 2013-11-12

**Authors:** Lorena Baboci, Paolo Boscolo-Rizzo, Dana Holzinger, Roberta Bertorelle, Lorena Biasini, Angelika Michel, Markus Schmitt, Giacomo Spinato, Rossana Bussani, Laia Alemany, Giancarlo Tirelli, Maria Cristina Da Mosto, Annarosa Del Mistro, Michael Pawlita

**Affiliations:** 1 Division of Genome Modifications and Carcinogenesis (F020), Research Program Infection and German Cancer Research Center (DKFZ), ImNeuenheimer Feld 280, Heidelberg 69120, Germany; 2 Department of Surgery, Oncology and Gastroenterology, Immunology and Oncology Section, University of Padua, Via Gattamelata, 64, Padua 35128-I, Italy; 3 Veneto Institute of Oncology IOV - IRCCS, Immunology and Molecular Oncology Unit, Via Gattamelata, 64, Padua 35128-I, Italy; 4 Department of Neurosciences, ENT Clinic and Regional Center for Head and Neck Cancer, University of Padua, School of Medicine, Treviso Regional Hospital, Piazzale Ospedale 1, Treviso 31100, Italy; 5 Head and Neck Department, Hospital of Cattinara, University of Trieste, Strada di Fiume 447, Trieste 34149, Italy; 6 Pathology Department, Hospital of Cattinara, University of Trieste, Strada di Fiume 447, Trieste 34149, Italy; 7 IDIBELL, Institut Catalàd’ Oncologia - Catalan Institute of Oncology, Avda Gran Via de l’Hospitalet, Barcelona 199-203, Spain

**Keywords:** HPV58, Head and neck squamous cell carcinoma, HPV carcinogenesis, HPV E6 and E7, HPV antibody, p16^INK4a^, pRb, p53

## Abstract

Persistent human papillomavirus infection (HPV) is recognized as an important etiologic factor for a subset of head and neck squamous cell carcinomas (SCC), especially those arising from the oropharynx. Whereas HPV16 accounts for the majority of HPV DNA-positive oropharyngeal SCC, infections with other mucosal high-risk HPV types are quite rare and biological data demonstrating their causal involvement are insufficient. Here we present the first case of an oropharyngeal SCC driven by HPV type 58. A 69-year-old Caucasian woman presented with an enlarged and firm left tonsil. A computed tomography scan showed a left tonsillar mass, extending to the soft palate and the glossotonsillar sulcus. The patient underwent extended radical tonsillectomy and ipsilateral selective neck dissection. Pathology confirmed an infiltrating, poorly differentiated SCC of the left tonsil with node metastasis (pT2N1). Adjuvant external beam radiation therapy (60 Grays (Gy)) was administered. After 1 year of follow-up, the patient is well with no evidence of cancer recurrence. HPV analyses of the tumor tissue by BSGP5+/6+ −PCR/MPG, targeting 51 mucosal HPV types, showed single positivity for HPV type 58. Presence of HPV58 E6*I RNA demonstrated biological activity of the virus in the tumor tissue, and presence of serum antibodies to HPV58 oncoproteins E6 and E7 indicated presence of an HPV58-driven cancer. Overexpression of cellular protein p16^INK4a^ and reduced expression of pRb, two cellular markers for HPV-induced cell transformation, were observed. Exons 4–10 of TP53 showed no mutations or polymorphisms. The presence of HPV58 as single HPV infection in combination with a broad variety of direct and indirect markers of HPV transformation provides comprehensive evidence that this oropharyngeal SCC was driven by HPV58.

## Background

Oropharyngeal squamous cell carcinomas (OPSCC) are categorized as head and neck squamous cell carcinoma (HNSCC) together with squamous cell carcinoma of the oral cavity, larynx and hypopharynx. OPSCC account for approximately 50,000 incident cases [1,2], and together with hypopharyngeal squamous cell carcinomas they account for about 1.1% of all malignancies worldwide [3]. Tobacco smoking and alcohol consumption are recognized as major risk factors but infection with Human papillomaviruses (HPV) has been identified as a causal factor for an increasing number of OPSCC, particularly in Waldeyer’s tonsillar ring [4,5].

Among the 51 mucosal HPV types known so far, 12 have been classified as carcinogenic (class I) for cervical cancer (CxCa) [6]. While HPV type 16 is the most prevalent type in CxCa worldwide (61%), the other carcinogenic types, i.e. HPV18, 31, 33, 35, 39, 45, 51, 52, 56, 58 and 59 (here referred to as non-HPV16 types) are responsible for approximately another 33% of CxCa, with HPV58 specifically accounting for 2% of CxCa. HPV58 in cervical cancer has the highest prevalence in Asia (4%), followed by North- and South-America (2% each), Europe (1%) and Africa (<1%) [7].

In contrast to CxCa, an even larger majority of HPV DNA-positive OPSCC are associated with HPV16 (89% - 97%), and DNA of other carcinogenic non-HPV16 types has been detected only rarely in OPSCC tissues [8-10]. A recent metanalysis of HPV DNA prevalence in head and neck cancers (Ndiaye C, Alemany L et al., in preparation) identified 11 (0.8%) HPV58 DNA positives among a total of 1466 HPV DNA positive oropharyngeal cancer cases that had been analysed for presence of HPV58 DNA [5,11-15].

Intriguingly, only a subset of HPV16 DNA-positive OPSCC display HPV16 carcinogenic activity in the tumor tissue, i.e. are HPV-driven (HPV DNA-positive RNA-positive) OPSCC, particularly in populations with low HPV DNA prevalence in OPSCC such as Western Europe. This indicates that presence of HPV DNA alone is not sufficient proof for causal involvement of any HPV DNA found in an OPSCC tissue. For non-HPV16 types found in OPSCC molecular proof of causality is largely lacking.

Over the last 25 years a rather detailed model of HPV-driven transformation of human tumor cells has been established. Truly HPV-transformed tumor cells contain at least one viral genome copy per cell and express the viral oncogenes E6 and E7. Interaction of the E6 and E7 oncoproteins with key regulators of cell cycle and apoptosis leads to upregulation of cellular protein p16^INK4a^ and downregulation of tumor suppressor proteins pRb and p53. In patients with invasive HPV-driven cervical, penile and oropharyngeal SCC overexpression of E6 and E7 oncoproteins frequently leads to strong antibody responses against these viral proteins [4,16-24].

This model has been extensively verified in cervical carcinoma for all HPV types classified by IARC/WHO as carcinogenic, probably and possibly carcinogenic [25]. It has also been fully or partially verified for HPV16 in squamous cell carcinoma of the oropharynx [8,26], larynx [27], oral cavity [28], penis [17] and anus [29].

Here, we present the first case of an OPSCC driven by HPV58, a carcinogenic HPV type with low prevalence in CxCa, as demonstrated by a broad variety of HPV transformation markers. The present case is part of an ongoing prospective multicentric study of head and neck cancer patients from North-East Italy.

## Case presentation

### Clinical data

A 69-year-old Caucasian woman presented with a 2 months history of foreign-body sensation in the throat. She had a negative history for tobacco and alcohol use. The patient underwent regular gynecological examinations and cytological cervical cancer screenings until the age of 64, and no HPV-associated genital lesions or cytological alterations were found. No anogenital tumor had become symptomatically apparent since then and extending also into the follow-up period after treatment of the index throat condition. The patient got infected with hepatitis B virus (HBV) by blood transfusion at the age of 26, and had been treated for lichen ruber planus in the oral cavity at the age of 34. The ENT (Ear Nose and Throat) physical examination revealed an enlarged left tonsil without ulceration. On palpation, the left tonsil was found to be firm to hard in consistency. She did not have any palpable cervical lymphadenopathy. A computed tomography (CT) scan with contrast of the neck confirmed a left tonsillar lesion of 20 × 23 × 26 mm in dimension, extending up to the soft palate, without crossing midline, and down to the glossotonsillar sulcus, and revealed two homogeneously enhancing lymph nodes, with maximum transverse diameters of 13 mm, in the left submandibular triangle. Baseline investigations including complete and differential blood cell count, serum electrolytes, liver and renal function tests, electrocardiogram, and X-ray chest, were well within normal limits. The patient underwent wedge biopsy of the left tonsillar mass. Pathologic review revealed the presence of a poorly differentiated SCC with basaloid features.

The patient underwent left side mandibulotomy, extended radical tonsillectomy and ipsilateral selective neck dissection (levels I-II-III-IV). The surgical defect was reconstructed with a microvascular ulnar forearm flap. Final pathology confirmed an infiltrating, poorly-differentiated SCC of the left tonsil, with tumor measuring 28 mm in greatest dimension and a thickness of 14 mm, abundant comedo-type necrosis and the presence of both vascular and perineural invasion. Histopathological examination of the neck dissection tissue showed an enlarged lymph node with intracapsular metastasis at level II (pT2N1). To minimize the risk of locoregional recurrence the treatment was completed with adjuvant external beam radiation therapy (EBRT), which started 7 weeks after surgery. Radiation treatment targeted the tonsillar bed and the left cervical region. A dose of 60 Gy was delivered with conventional fractionation (2 Gy/fraction, once daily, five times weekly). The HPV molecular data were not available at the time of clinical diagnosis and tumor treatment. After 1 year of follow-up, the patient is well with no evidence of recurrent cancer.

### Molecular analyses

Analyses were performed in fresh-frozen tumor biopsy, formalin-fixed paraffin-embedded (FFPE) tumor tissue and serum. All samples were collected at the time of initial tumor diagnosis before surgery and radiotherapy.

Genomic DNA was extracted from sections of fresh-frozen and FFPE tissues following previously described procedures [8,25,30]. More than 80% of the cells in hematoxylin eosin-stained adjacent sections were neoplastic.

HPV genotyping was performed by BSGP5+/6+ −PCR/MPG [31-33], an assay capable of amplifying about 150 base pairs (bp) of the L1 gene of 51 HPV mucosal types, followed by hybridization to type-specific probes on fluorescent suspension array beads. It includes detection of the human β-globin gene as DNA quality control as well as internal PCR and hybridization controls. Sample DNA was also analyzed by MY09/11-PCR amplification followed by both Restriction Fragment Length Polymorphism (RFLP) analysis and direct sequencing [34].

Genomic DNA extracted from both fresh-frozen and FFPE tumor tissues contained HPV58 as single mucosal HPV infection, as shown by both mucosal HPV genotyping methods.

Total RNA was obtained from FFPE tissue sections using the Pure-Link FFPE Total RNA Isolation Kit (Invitrogen, Carlsbad, CA) following the manufacturer’s protocol. Viral RNA expression was analyzed by E6*I reverse transcription (RT)-PCR [25]. Briefly, the HPV type-specific assay generates 70 bp long cDNA amplicons across the E6*I splice site that are subsequently detected by hybridization to type-specific and splice site-specific probes on fluorescent suspension array beads. A duplex assay was performed for RNA of HPV16 and the cellular housekeeping gene ubiquitin C (UbC), and a singleplex assay for HPV58. The sample was positive for the HPV58 E6*I and UbC transcripts and negative for the HPV16 E6*I transcript.

**Figure 1:**
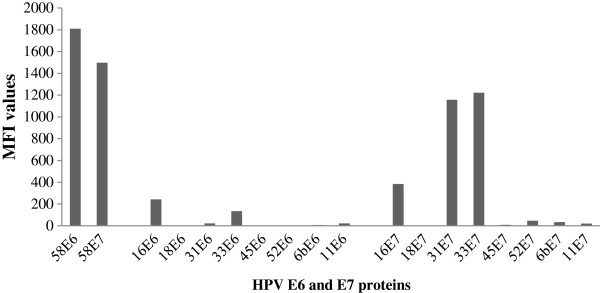
**E6 and E7 antibodies status for HPV58 and other HPV types (16, 18, 31, 33, 45, 52, 6 and 11).** MFI (Mean Fluorescence Intensity) values (y axis) are shown for the early proteins E6 and E7 of HPV58 and other HPV types (16, 18, 31, 33, 45, 6, 11) (x axis). Left: E6 and E7 MFI values for HPV58; Center and right: the MFI values for the other HPV types tested, grouped as E6 and E7, respectively.

The HPV antibody status was analyzed by a glutathione S-transferase (GST) capture immunosorbent assay using as antigens full-length HPV proteins bacterially expressed as GST fusion proteins [35,36] in combination with fluorescent suspension array beads as previously described [4,37,38]. Antibodies to the E6 and E7 oncoproteins of high-risk HPV types 16, 18, 31, 33, 45, 52 and 58 and low-risk types 6 and 11 were quantified. Positivity was high for E6 and E7 of HPV58. Weaker signals were observed for E7 of the closely related HPV types 31 and 33 and are interpreted as cross-reactivity with the HPV58 E7 protein (Figure 1).

**Figure 2:**
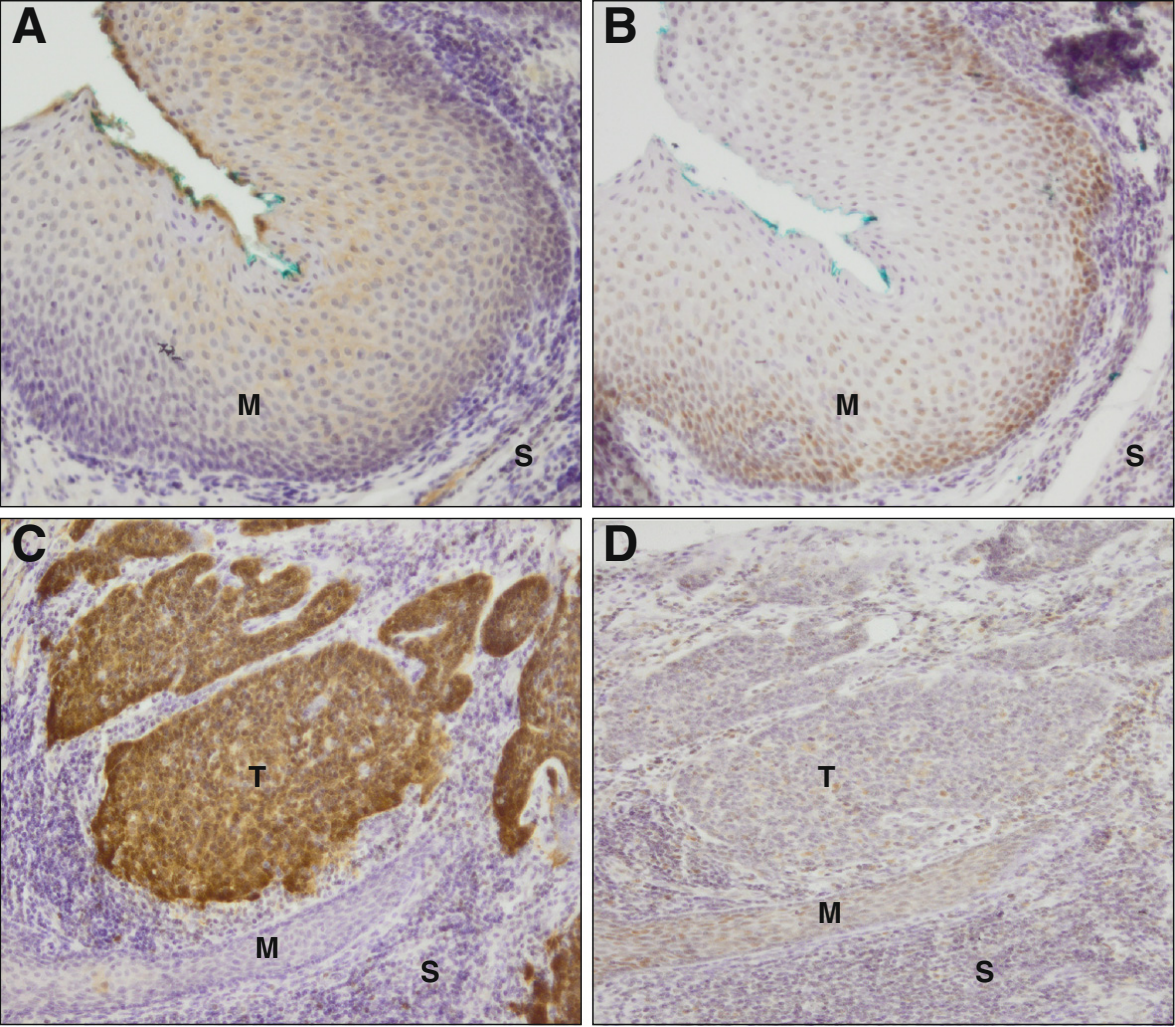
**Immunohistochemistry of cellular proteins p16**^**INK4a **^**and pRb. A** and **C**, p16^INK4a^ expression in normal tumor-adjacent mucosa (M) and in tumor (T) tissue, respectively. **B** and **D**, pRb expression in normal tumor-adjacent mucosa and in tumor tissue, respectively. S, stroma. Original magnification 10×. p16^INK4a^ low in the normal tumor-adjacent mucosa (blue-stained nuclei **(A)**), high in the tumor (brown stained nuclei **(C)**). pRb high in normal tumor-adjacent mucosa (brown-stained nuclei **(B)**), low in the tumor (blue-stained nuclei **(D)**).

The expression level of the cellular proteins p16^INK4a^ and pRb, markers for HPV transformation, were evaluated by immunohistochemistry (IHC). The monoclonal antibodies CINtec (V-kit, MTM laboratories, Heidelberg, Germany) and NCL-RB (Novocastra, Newcastle, UK), were used for p16^INK4a^ and pRb, respectively. Well characterized sections from CxCa and a healthy mucosa were used in each staining batch as reference for scoring of the protein expression levels for both markers. IHC was evaluated independently by two investigators. The tumor cells of the sections showed strong nuclear and diffuse cytoplasmic staining of p16^INK4a^ (>95%) and low staining of pRb (<25%), while proliferating basal and parabasal cells of the tumor-adjacent mucosa showed low p16^INK4a^ (<5%) and high pRb (>25%) expression (Figure 2).

TP53 mutation status was determined in genomic DNA isolated from the fresh-frozen biopsy. Exons 4–10 were amplified using specific primer pairs for each exon as described in the IARC protocol [39]. Amplification products were sequenced using the ABI PRISM® 3730XL Genetic Analyzer (Applied Biosystems, Foster City, CA). No mutations or polymorphisms were observed.

## Discussion

HPV-driven OPSCC appears to represent a tumor entity distinct from HPV-negative OPSCC. Patients with HPV-driven OPSCC in comparison to patients with HPV-negative OPSCC have a better outcome and overall survival, independent of treatment regimen [40,41]. These tumors also have been shown to contain fewer overall somatic mutations/genetic changes [42]. Furthermore, while in HPV-negative OPSCC tumor suppressor TP53 mutations are present in up to 100%, such mutations are absent in HPV-positive OPSCC [42-44]. It therefore appears to be clinically important for prognosis and potentially for choice of treatment options to determine the HPV-status of OPSCC cases.

While for HPV16, the HPV-type most frequently found in HPV DNA-positive OPSCC, a transforming role in the pathogenesis of a subgroup of OPSCC has been thoroughly demonstrated [8,16,45], this evidence is largely lacking for non-16 HPV types that are found in only 3-14% of OPSCC by HPV genotyping studies applying sensitive PCR techniques [9,10]. However, as demonstrated for HPV16 in several studies, presence of HPV DNA alone is not sufficient evidence for causal involvement of the HPV type found [8,22,45-47].

The HPV58-positive OPSCC case we describe here is, to our knowledge, the first case for which ample molecular and serological evidence is provided to demonstrate causal involvement of this rare high-risk HPV type in the pathogenesis of this carcinoma. In addition to the presence of HPV DNA in the tumor tissue, we demonstrate (i) presence of HPV58 E6*I RNA encoding the viral oncoprotein E7, which in HPV58-positive cervical carcinoma is a consistent marker of biological activity of the virus [27]; (ii) strong serum antibody response to both HPV58 oncoproteins E6 and E7; HPV type concordant E6 and E7 double antibody positivity has been demonstrated to be highly associated and specific for invasive HPV-associated cancers with an extremely low prevalence of about 0.1% in tumor-free individuals [4,18,21] and to be strongly associated (odds ratios of 44 to 180) with invasive cancer of the cervix [20], the penis [17] and the upper aerodigestive tract [4,18,21]; (iii) overexpression of p16^INK4a^ and reduced expression of pRb, cellular markers for HPV transformation strongly associated with HPV16-transformed OPSCC [26] and also HPV58-transformed cervical cancers [25,48], and finally (iv) wildtype sequence of TP53, a marker strongly associated with HPV16-positive OPSCC [42]. We further excluded the possibility of HPV16 involvement by demonstrating absence of HPV16 DNA, of HPV16 E6*I RNA and of HPV16 E6 and E7-specific antibodies. However, due to the rare involvement of HPV58 in HNSCC, multicentric studies are needed to evaluate the survival of the patient(s) to confirm the better prognosis like it was shown for HPV16-positive cases.

## Conclusion

This HPV58-driven oropharyngeal carcinoma is the first well-documented case of HPV transformation in the oropharynx by a non-16 HPV-type.

### Consent

Written informed consent was obtained from the patient for publication of this Case Report and any accompanying images. A copy of the written consent is available for review by the Editor-in-Chief of this journal.

## Abbreviations

CxCa: Cervical carcinoma; ENT: Ear, nose and throat; FFPE: Formalin-fixed paraffin-embedded; GST: Gluthatione S-transferase; Gy: Gray; HPV: Human papillomavirus; IARC: International agency for research on cancer; IHC: Immunohistochemistry; OPSCC: Oropharyngeal squamous cell carcinoma; PCR: Polymerase chain reaction; pRb: Retinoblastoma protein; UbC: Ubiquitin C; WHO: World Health Organization.

## Competing interests

The authors L. Baboci, D. Holzinger, P. Boscolo-Rizzo, A. Del Mistro, G. Spinato, R. Bertorelle, L. Biasini, A. Michel, R. Bussani, L. Alemany, G. Tirelli and M.C. Da Mosto disclosed no potential conflicts of interest. M. Schmitt and M. Pawlita have received research support through cooperation contracts of DKFZ with Roche and Qiagen in the field of development of HPV diagnostics.

## Authors’ contributions

LB carried out the molecular analyses and drafted the manuscript, MP conceived the study, participated in its design and coordination, and supervised to draft the manuscript, PBR, GS, GT, RB and MCDM participated in the management of the patient, provided the samples and contributed to draft the clinical part of the manuscript, ADM contributed and supervised to draft the manuscript, DH supervised the molecular analyses and contributed to draft the manuscript, LB and RB performed the TP53 sequencing analyses, AM provided the serological data. MS contributed to draft the manuscript. LA provided unpublished metanalyses data on HPV type 58. All authors read and approved the final manuscript.
